# Stratification Tools for Disease‐Modifying Trials in Prodromal Synucleinopathy

**DOI:** 10.1002/mds.28785

**Published:** 2021-09-17

**Authors:** Dario Arnaldi, Pietro Mattioli, Francesco Famà, Nicola Girtler, Andrea Brugnolo, Matteo Pardini, Andrea Donniaquio, Federico Massa, Beatrice Orso, Stefano Raffa, Matteo Bauckneht, Silvia Morbelli, Flavio Nobili

**Affiliations:** ^1^ Department of Neuroscience (DINOGMI) University of Genoa Genoa Italy; ^2^ IRCCS Ospedale Policlinico San Martino Genoa Italy; ^3^ Department of Health Sciences (DISSAL) University of Genoa Genoa Italy

**Keywords:** SPECT, EEG, cognitive, prodromal synucleinopathy, REM sleep behavior disorder

## Abstract

**Background:**

Dopamine transporter single photon‐emission computed tomography (DAT‐SPECT) is the strongest risk factor for phenoconversion in patients with idiopathic rapid eye movement (REM)‐sleep behavior disorder (iRBD). However, it might be used as a second‐line stratification tool in clinical trials, because it is expensive and mini‐invasive.

**Objective:**

Aim of the study is to investigate whether other cost‐effective and non‐invasive biomarkers may be proposed as first‐line stratification tools.

**Methods:**

Forty‐seven consecutive iRBD patients (68.53 ± 7.16 years, 40 males) underwent baseline clinical and neuropsychological assessment, olfaction test, resting electroencephalogram (EEG), and DAT‐SPECT. All patients underwent 6 month‐based clinical follow‐up to investigate the emergence of parkinsonism and/or dementia. Survival analysis and Cox regression were used to estimate conversion risk.

**Results:**

Seventeen patients developed an overt synucleinopathy (eight Parkinsonism and nine dementia) 32.8 ± 22 months after diagnosis. The strongest risk factors were putamen specific to non‐displaceable binding ratio (SBR) (hazard ratio [HR], 7.3), attention/working memory cognitive function (NPS‐AT/WM) (HR, 5.9), EEG occipital mean frequency (HR, 2.7) and clinical motor assessment (HR, 2.3). On multivariate Cox‐regression analysis, only putamen SBR and NPS‐AT/WM significantly contributed to the model (HR, 6.2, 95% confidence interval [CI], 1.9–19.8). At post‐hoc analysis, the trail‐making test B (TMT‐B) was the single most efficient first‐line stratification tool that allowed to reduce the number of eligible subjects to 76.6% (sensitivity 1, specificity 0.37). Combining TMT‐B and DAT‐SPECT further reduced the sample to 66% (sensitivity 0.88, specificity 0.47).

**Conclusion:**

The TMT‐B seems to be a cost‐effective and efficient first‐line screening tool, to be used to select patients that deserve DAT‐SPECT as second‐line screening tool for disease‐modifying clinical trials. © 2021 The Authors. *Movement Disorders* published by Wiley Periodicals LLC on behalf of International Parkinson and Movement Disorder Society

Several disease‐modifying drugs for synucleinopathies, in particular for Parkinson disease (PD), are now being developed and tested. Moreover, disease‐modifying trials are now in development, although not ongoing yet, for prodromal synucleinopathies too, with the expectation of increasing the efficacy of such drugs by intercepting pathology at earliest stages. Patients suffering from idiopathic/isolated rapid eye movement (REM) sleep behavior disorder (iRBD) are likely the readiest neuroprotective trial cohort in this field. However, the phenoconversion time of iRBD patients can be longer than 10 years,^1^ and therefore, risk factors for short‐term phenoconversion are needed.

To achieve an efficient stratification of eligible prodromal synucleinopathy patients, reliable biomarkers are urgently needed. First‐line stratification tools should be cost‐ and time‐effective, non‐invasive, widely available, and possibly, largely validated because those tools will be administered to large numbers of eligible patients with the aim of reducing the number of subjects that will undergo second‐line screening tools. Ideally, first‐line stratification tools should also have nearly perfect sensitivity to ensure not to exclude potential phenoconverters. On the other hand, second‐line stratification tools should reliably identify patients with high risk of short‐term phenoconversion with high accuracy, and can be more invasive and less cost‐effective because they will be administered to selected eligible subjects only. Ideally, second‐line stratification tools should also monitor disease progression. Indeed, a clinical trial stratification process should include patients at high risk of short‐term conversion, but also exclude patients that are likely non‐converters or late‐converters.

The first step for identifying eligible prodromal synucleinopathy patients will be having a polysomnography‐confirmed iRBD. Several risk factors for phenoconversion in iRBD patients have been proposed, including hyposmia, mild cognitive or motor impairment, dysautonomia, and presynaptic dopaminergic impairment.^1^, ^2^ However, multivariate studies investigating the best first‐ and second‐line stratification tools to identify eligible iRBD patients to be enrolled in the upcoming disease‐modifying trials are still lacking.

The aim of the present study was to investigate clinical, neuropsychological, neurophysiological, and functional brain imaging features of a group of iRBD patients to define which factor is more suitable as a first‐line stratification tool to reduce the number of eligible patients that would undergo second‐line tools, in view of an enrollment strategy for upcoming disease‐modifying studies in prodromal synucleinopathies.

## Methods

### Patients

Forty‐seven consecutive iRBD patients admitted to our university hospital‐based sleep unit between 2012 and 2018 were prospectively enrolled. The main inclusion criteria were age over 50 and the presence of video‐polysomnography‐confirmed RBD.^3^ The main exclusion criteria were history of major neurological or psychiatric disease, including but not limited to parkinsonism and dementia, therefore, defining the patients as iRBD. Patients with uncontrolled systemic diseases, including but not limited to diabetes, organ failure, and tumors were excluded. Patients were either not taking antidepressants or they underwent a 4‐week withdrawal period before video‐polysomnography. Brain magnetic resonance imaging (MRI), or computed tomography in the case MRI was unfeasible, was used to rule out brain diseases. The presence of white matter hyperintensities was not an exclusion criterion if the Whalund scale was not >1 for each brain region.^4^

Patients underwent baseline clinical evaluation, including (1) the Movement Disorder Society‐sponsored revision of the unified Parkinson's disease rating scale, motor section (MDS‐UPDRS‐III) to evaluate the presence of parkinsonism; (2) clinical interview and questionnaires for activities of daily living (ADL) and instrumental ADL to exclude dementia; (3) Beck depression inventory II (BDI‐II) to rate depressive symptoms; (4) blood pressure measurement in supine and after 3 minutes of standing to assess orthostatic hypotension; (5) clinical interview to assess constipation; (6) the Smell Diskettes Olfaction test^5^ or the Sniffin' Sticks Test^6^ to assess olfaction. Moreover, patients underwent a comprehensive neuropsychological assessment, evaluating the main neuropsychological domains (ie, language, executive functions, visuospatial abilities, memory, attention, and working memory), including semantic and phonemic verbal fluency, Stroop color word, Stroop color, Trail making test (TMT) A and B, clock completion, constructional apraxia (simple copy and copy with guiding landmarks), Rey Auditory Verbal Memory Test (RAVLT, immediate and delayed recall), Babcock story, Corsi span, digit span, and symbol digit. References for tests and normative values are listed in a previous paper.^7^ The presence of mild cognitive impairment (MCI) was evaluated according to the PD‐MCI criteria, by level two assessment.^8^

All patients underwent 6 month‐based clinical follow‐up, after the baseline assessment, to investigate the emergence of signs/symptoms of parkinsonism (defined as bradykinesia plus at least one of rigidity or rest tremor)^9^ and/or dementia (defined as functional impairment in instrumental ADL and with evidence of cognitive impairment on standardized testing).^10^ Patients who develop parkinsonism and/or dementia at follow‐up were considered converters for statistical analysis, whereas the remaining patients were considered as non‐converters. Diagnosis of PD and dementia with Lewy bodies (DLB) were made at the follow‐up visit, according to current criteria.^9^, ^11^ The so‐called 1‐year rule was used for patients who developed both parkinsonism and dementia.^11^

The study protocol met the approval of the local Ethics Committee and all participants signed an informed consent form in compliance with the Helsinki Declaration of 1975.

### [ ^123^I]‐Ioflupane SINGLE‐PHOTON EMISSION COMPUTED TOMOGRAPHY

Within 3 months of the baseline assessment, patients underwent [123I]‐ioflupane single‐photon emission computed tomography (^123^I‐FP‐CIT‐SPECT) according to the European Association of Nuclear Medicine guidelines^12^ to evaluate nigrostriatal dopaminergic functioning. Specific to non‐displaceable binding ratios (SBRs) at putamen and caudate levels were computed^13^ and transformed into *z* scores, adjusted for age, based on the European normative database of 122 healthy subjects,^14^ as detailed in a previous paper.^15^ ^123^I‐FP‐CIT images were exported into an analyze format and processed by the automatic BasGan algorithm^13^ based on a high‐definition, three‐dimensional striatal template derived from the Talairach atlas, using the occipital lobes uptake as the background reference region. Inter‐hemispheric mean values of caudate and putamen SBR were used in statistical analysis.

### EEG Recording and Data Processing

Within 1 month of baseline assessment, a scalp electroencephalogram (EEG) was recorded during relaxed wakefulness, as detailed in a previous paper.^16^ Patients were not taking benzodiazepines at the time of EEG. Quantitative EEG (qEEG) relative band power for each conventional frequency band (ie, delta, theta, alpha, sigma, and beta) and mean frequency (MF) values in the 2–16 Hz band were computed in frontal (Fp1/F3, Fp1/F7, Fp2/F4, Fp2/F8, and Fz/Cz), centro‐parietal (F3/C3, C3/P3, F4/C4, C4/P4, and Cz/Pz), temporal (F7/T3, T3/T5, F8/T4, and T4/T6), and occipital (P3/O1, T5/O1, P4/O2, and T6/O2) regions. Finally, the ratio between α (8–12 Hz) and θ (4–8 Hz) band power (α/θ ratio) in each region was calculated to obtain a measure of background activity to be used in statistical analyses.

### Healthy Controls

Three control groups were set up with a case–control criterion, matched for gender and age. General medical history, clinical, and neurologic examination were carefully reviewed to confirm their healthy condition. A first control group for neuropsychological tests (HC1) included 40 healthy controls (70.03 ± 8.15 years; education 12.1 ± 4.0 years; 17 males) undergoing the same neuropsychological evaluation of patients. A second control group was set for ^123^I‐FP‐CIT‐SPECT (HC2) and comprised of 53 healthy controls (69.68 ± 7.99 years; 46 males). A third control group was set for qEEG (HC3) comprised of 44 healthy controls (age 69.55 ± 8.08; 30 males).

### Statistical Analysis

As a first descriptive step, neuropsychological, ^123^I‐FP‐CIT‐SPECT, and qEEG data were compared between iRBD and related control groups.

Normal distribution of variables were checked using Shapiro–Wilk test. Continuous variables were compared using unpaired *t* test (normally distributed) or the Wilcoxon‐Mann–Whitney test (non‐normally distributed). Categorical variables were compared using χ^2^ or Fisher's exact tests. The Bonferroni method was used to correct for multiple comparisons.

To minimize multicollinearity and to reduce the number of neuropsychological variables for further statistical analysis, factor analysis with varimax rotation was applied to the baseline native neuropsychological measures to identify the variables expressing a similar part of total variance. A conventional threshold of 0.4 was applied to factor loadings (expressing the factor‐variable correlation) to identify the group of variables mainly represented by each factor.

Subsequently, Kaplan–Meier survival analysis was performed to estimate conversion risk on the ^123^I‐FP‐CIT‐SPECT, neuropsychological, qEEG, and clinical features. Continuous variables were categorized as above or below a cut‐point identified by the Youden method. Censoring time was set at the time of last assessment for non‐converters and at the time of conversion for converter patients. Hazard ratios (HRs) for each variable were calculated with Cox regression. A Cox regression model was applied with a forward stepwise approach, adjusting for age, to identify the best combination of predictors for phenoconversion. Even if it is not strictly necessary that the best predictors are independent, we choose this approach to reduce the number of variables and retain only the most relevant ones for subsequent analysis. However, we also provide the HR and area under the curve (AUC) values for all significant variables in Tables S1 and S2 in Supplementary Appendix S1.

Finally, a receiver operating characteristic (ROC) analysis was performed to investigate whether baseline ^123^I‐FP‐CIT‐SPECT, neuropsychological, qEEG, and clinical data were able to predict the phenoconversion diagnosis (ie, PD vs. DLB converters). The limited number of phenoconverted patients did not allow a more detailed statistical analysis.

Statistical analyses were performed using Stata software (StataCorp, 2013, Stata Statistical Software: Release 13. College Station, TX).

## Results

Table 1 summarizes main demographic and clinical data of iRBD patients and the three control groups. As per the selection criteria, there were no significant differences in demographic data.

**TABLE 1:** Demographic and clinical data of iRBD patients and the three healthy control groups

	iRBD	HC1	HC2	HC3	*P* value
N (male)	47 (40)	40 (37)	53 (46)	44 (37)	0.672
Age (y)	68.5 ± 7.2	70.0 ± 8.2	69.7 ± 7.9	69.6 ± 8.0	0.325
Education (y)	10.5 ± 4.3	12.1 ± 4.0			
MMSE	28.5 ± 1.5	29.2 ± 0.9			
BDI‐II	11.3 ± 8.3	12.6 ± 8.4			
MCI	18 (38%)	0 (0%)			
MDS‐UPDRS‐III	0.83 ± 1.8				
Hyposmia	24 (51%)				
Constipation	23 (49%)				
Orthostatic hypotension	7 (15%)				
Neuropsychological data					
Categorical verbal fluency	40.2 ± 9.5	43.9 ± 12.3			0.056
Phonological verbal fluency	31.0 ± 10.7	35.5 ± 9.3			0.021*
Stroop color	38.1 ± 12.4	43.0 ± 10.0			0.023*
Stroop color word	15.4 ± 8.1	19.6 ± 6.4			0.005*
Digit span	5.6 ± 1.0	5.9 ± 1.1			0.110
Corsi span	4.8 ± 1.1	4.9 ± 0.9			0.341
TMT A	59.2 ± 25.0	51.2 ± 22.4			0.062
TMT B	163.1 ± 117.0	116.9 ± 68.8			0.015*
TMT B‐A	103.9 ± 97.8	65.7 ± 57.4			0.016*
Symbol digit	30.6 ± 12.8	38.8 ± 10.1			<0.001*
CDT	13.0 ± 3.0	14.8 ± 0.7			<0.001*
CA simple copy	9.2 ± 1.8	9.8 ± 1.1			0.023*
CA copy with guiding landmarks	66.6 ± 5.4	68.7 ± 1.8			0.014*
Rey immediate recall	33.8 ± 1.9	40.2 ± 10.2			0.001*
Rey delayed recall	6.6 ± 2.8	8.2 ± 3.2			0.008*
Babcock story recall	12.1 ± 3.8	15.8 ± 3.7			<0.001*
^123^I‐FP‐CIT‐SPECT data					
Left caudate SBR	3.36 ± 0.98		4.41 ± 1.06		<0.001*
Right caudate SBR	3.32 ± 0.97		4.31 ± 0.98		<0.001*
Left putamen SBR	2.63 ± 0.97		3.51 ± 0.84		<0.001*
Right putamen SBR	2.71 ± 0.96		3.67 ± 0.94		<0.001*
qEEG data					
Frontal α/θ ratio	1.23 ± 0.93			1.78 ± 1.08	0.005*
Temporal α/θ ratio	1.90 ± 1.83			4.22 ± 2.89	<0.001*
Centro‐parietal α/θ ratio	2.78 ± 3.37			4.46 ± 2.89	0.006*
Occipital α/θ ratio	2.47 ± 2.65			6.39 ± 5.68	<0.001*
Frontal MF (Hz)	10.14 ± 1.96			8.92 ± 1.75	0.002*
Temporal MF (Hz)	10.29 ± 2.09			9.89 ± 0.94	0.132
Centro‐parietal MF (Hz)	10.05 ± 1.99			10.31 ± 1.28	0.235
Occipital MF (Hz)	9.76 ± 1.88			10.30 ± 0.91	0.050*

Data are shown as mean ± standard deviation.

Abbreviations: BDI, Beck depression inventory II; CA, constructional apraxia; MCI, mild cognitive impairment; MDS‐UPDRS‐III, MDS revision of the unified Parkinson's disease rating scale, motor section; MMSE, mini mental state examination; MF, mean frequency; SBR, specific to non‐displaceable binding ratio; TMT, trail making test.

* Significant *P* values.

As expected, iRBD patients showed impaired cognitive functions, mainly in verbal memory and visuospatial abilities, a diffuse reduction of nigrostriatal dopaminergic activity and reduced qEEG features, especially the α/θ ratios, compared with healthy controls.

### Factor Analysis

Factor analysis identified five factors (Table 2). Factor one was mainly related to attention and working memory (NPS‐AT/WM), factor two to verbal memory (NPS‐VM), factor three to visuospatial abilities (NPS‐VS), factor four to executive functions (NPS‐EX), respectively. Factor five included two tests (phonemic fluency and digit span) also belonging to executive functions, and it was named NPS‐EX2.

**TABLE 2:** Factor analysis results

	NPS‐AT/WM	NPS‐VM	NPS‐VS	NPS‐EX	NPS‐EX2
TMT‐A	−0.76				
TMT‐B	−0.95				
Symbol digit	0.58				
Corsi span	0.57				
Semantic verbal fluency	0.54				
RAVLT, immediate recall		0.82			
RAVLT, delayed recall		0.77			
Babcock story		0.60			
Clock completion test			0.47		
CA simple copy			0.66		
CA guiding landmarks			0.66		
Stroop color				0.70	
Stroop color word				0.73	
Phonemic verbal fluency					0.57
Digit span					0.66
Variance explained (%)	36.8	21.5	15.9	14.3	9.7

Factors and corresponding neuropsychological tests with their respective factor loading are shown. A conventional factor loading threshold of 0.4 was used.

Abbreviations: CA, constructional apraxia; RAVLT, Rey auditory verbal memory test; TMT, trail making test.

### Survival Analysis

Mean follow‐up was 37 ± 18 months from diagnosis. Seventeen patients (36.2%) developed a full‐blown neurodegenerative disease, with a mean conversion time of 32.8 ± 22 months after diagnosis. Eight patients developed parkinsonism first, receiving a PD diagnosis (conversion time 30 ± 29.6 months) and nine developed dementia first, receiving a DLB diagnosis (conversion time 35 ± 13.2 months). Two of the eight PD‐converters subsequently developed dementia, 2 and 3 years after parkinsonism onset, respectively, and were, therefore, labeled as affected by PD dementia. All DLB‐converters also developed parkinsonism within 1 year from dementia diagnosis.

**FIG 1:**
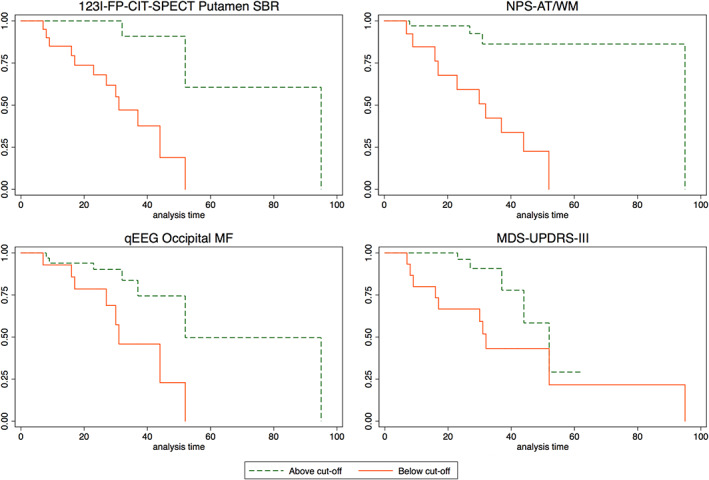
Kaplan–Meier disease‐free survival plot of iRBD patients according to the best predictors of phenoconversion. For each feature, red solid lines indicate patients below cut‐off and green dashed lines indicate patients above cut‐off. [Color figure can be viewed at wileyonlinelibrary.com]

All investigated variables were significant predictors of future phenoconversion (*P* < 0.05, see Table S1 in Supplementary Appendix S1). On Cox proportional hazards analysis (Fig. 1), the best (ie, the one with the highest HR) features in predicting phenoconversion were putamen SBR (cut‐off, −1.5 *Z* score; HR, 7.3, 95% confidence interval [CI], 1.8–29.4) for ^123^I‐FP‐CIT‐SPECT data, NPS‐AT/WM (cut‐off, 0.17; HR, 5.9, 95% CI, 1.8–19.7) for neuropsychological data, occipital MF (cut‐off, 9 Hz; HR, 2.8, 95% CI, 1.0–7.8) for qEEG data, and MDS‐UPDRS‐III (cut‐off, 1; HR, 2.3, 95% CI, 0.8–6.2) for clinical data. In the subsequent forward stepwise Cox proportional hazard analysis, only putamen SBR and NPS‐AT/WM significantly contributed the model (HR, 6.2, 95% CI, 1.9–19.8). Adding other variables did not significantly improve the model. All analyses were adjusted for age.

Considering that only putamen SBR and NPS‐AT/WM were retained in the final model, a post hoc analysis was conducted to find the best cognitive risk factors of phenoconversion to be used as stratification markers in clinical trials. The TMT‐B was by far the most important feature of the NPS‐AT/WM factor (Table 2). Therefore, a ROC analysis was performed on TMT‐B, and a TMT‐B cut‐off of 85 seconds correctly identified all phenoconverters (sensitivity 1, specificity 0.37), whereas 19 among the 30 (63.3%) non‐phenoconverters had a TMT‐B above the 85 second cut‐off. Using this cut‐off as the first‐line stratification tool would reduce the number of eligible subjects to 36 (76.6%). Among these 36 patients, by using a *Z* score cut‐off of −1.5 for putamen SBR, 11 of 17 (64.7%) converters and only 4 of 19 (21.1%) non‐converters were identified. Using instead a −1 *Z* score cut‐off led to identify 15 of 17 (88.2%) converters, but also 16 of 19 (84.2%) non‐converters.

Conversely, a TMT‐B cut‐off of 172 seconds (“best cut‐off” according to the Youden method) correctly identified 12 of the 17 phenoconverters (sensitivity 0.71, specificity 0.93), but only 2 among the 30 (7%) non‐phenoconverters, therefore, reducing the sample to 14 (29.8%).

**FIG 2:**
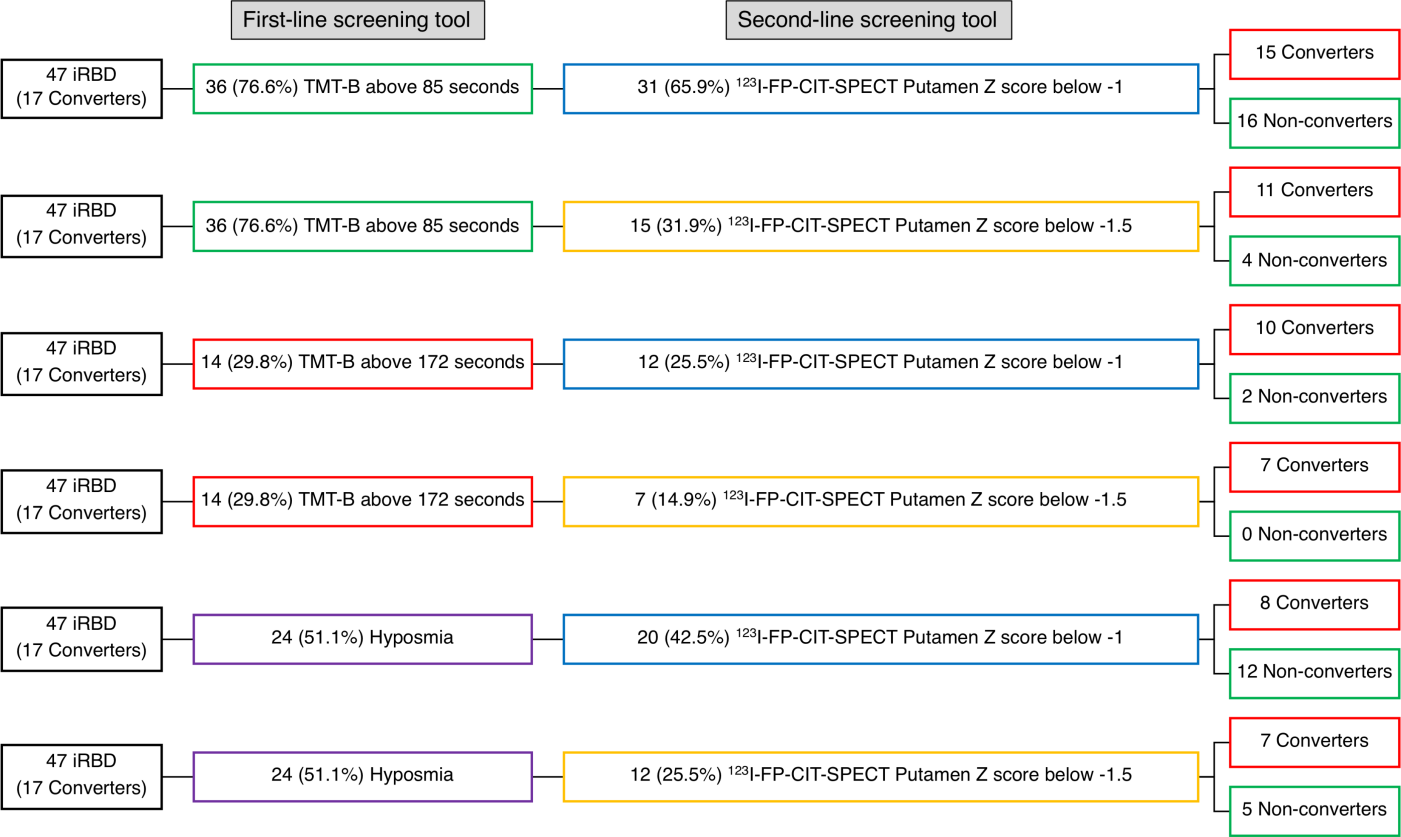
The stratification procedure of iRBD patients using trail‐making part B (TMT‐B) or hyposmia as the first‐line stratification tool and ^123^I‐FP‐CIT‐SPECT as the second‐line stratification tool. In brackets, the percentage of stratified patients out of the starting sample (47 iRBD patients) is reported. [Color figure can be viewed at wileyonlinelibrary.com]

For comparison, hyposmia was present in 9 of 17 phenoconverters (52.9%) and in 15 among non‐phenoconverters (50%). The detailed stratification procedure is detailed in Figure 2.

The combination of TMT‐B above 85 seconds and ^123^I‐FP‐CIT putamen *Z* score below −1 had a sensitivity of 0.88 and a specificity of 0.47 (accuracy 0.62) in distinguishing between phenoconverters and non‐phenoconverters. The combination of TMT‐B above 85 seconds and ^123^I‐FP‐CIT putamen *Z* score below −1.5 had a sensitivity of 0.65 and a specificity of 0.87 (accuracy 0.79). The combination of TMT‐B above 172 seconds and ^123^I‐FP‐CIT putamen *Z* score below −1 had a sensitivity of 0.59 and a specificity of 0.93 (accuracy 0.81). The combination of TMT‐B above 172 seconds and ^123^I‐FP‐CIT putamen *Z* score below −1.5 had the best specificity (1.0) but with a low sensitivity (0.41, accuracy 0.79). For comparison, the combination of hyposmia and ^123^I‐FP‐CIT putamen *Z* score below −1 had 0.47 sensitivity with 0.60 specificity (accuracy 0.55), whereas the combination of hyposmia and ^123^I‐FP‐CIT putamen *Z* score below −1.5 had 0.41 sensitivity with 0.83 specificity (accuracy 0.68).

### PD‐Converters Versus DLB‐Converters Patients

**FIG 3:**
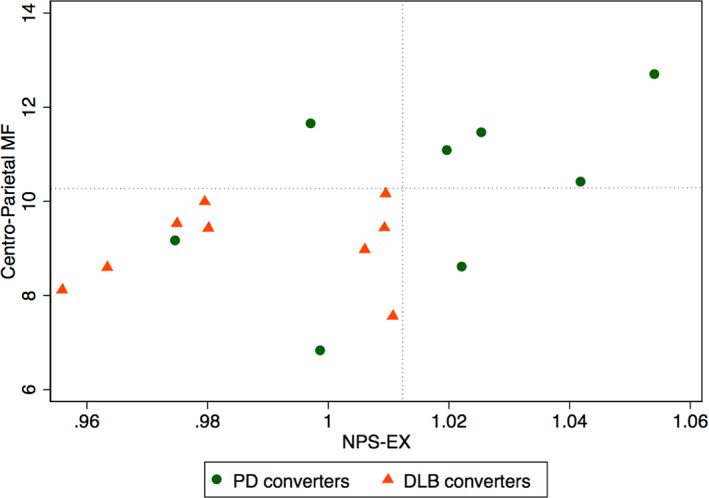
Scatter plot of centro‐parietal MF versus NPS‐EX data of iRBD patients. The dotted lines indicate the cut‐off used for ROC analysis. Green circles indicate PD converters and red triangles DLB converters. [Color figure can be viewed at wileyonlinelibrary.com]

The ROC analysis showed that, compared with PD‐converters, DLB‐converters had reduced NPS‐EX (AUC, 0.81), lower centro‐parietal MF (AUC, 0.81) and reduced caudate SBRs (AUC, 0.71). NPS‐EX and centro‐parietal MF values in phenoconverted iRBD patients are shown in Figure 3. All DLB‐converters are below both centro‐parietal MF and NPS‐EX cut‐off values (sensitivity 1) and only two PD converted patients are below those two cut‐offs (specificity 0.75). Interestingly, one of these two patients further developed dementia after ~3 years of PD diagnosis, whereas the other had MCI since iRBD diagnosis. Moreover, in phenoconverted iRBD patients, NPS‐EX, and centro‐parietal MF are significantly correlated with each other (r = 0.51, *P* = 0.035), whereas no significant correlation was found between the two variables in non‐converters.

## Discussion

In this study, we explored the combined use of clinical, functional brain imaging, neuropsychological, and, for the first time, EEG markers in predicting the short‐term phenoconversion of iRBD patients, with the aim of identifying first‐ and second‐line stratification tools for upcoming disease‐modifying clinical trials. First, we found that the nigroputaminal dopaminergic function, as investigated by ^123^I‐FP‐CIT‐SPECT, is the single, most reliable risk factor for future phenoconversion, within 3 years from diagnosis, in agreement with literature. Indeed, iRBD patients often have nigrostriatal dopaminergic impairment.^17^ Moreover, several single‐center studies found that presynaptic dopaminergic imaging is a good predictor of subsequent phenoconversion of iRBD patients.^18^, ^19^, ^20^, ^21^ Finally, a recent international multicenter study, found that nigroputaminal dopaminergic impairment, as investigated by ^123^I‐FP‐CIT‐SPECT, is the strongest predictor of phenoconversion in iRBD.^2^ Therefore, ^123^I‐FP‐CIT‐SPECT is likely going to be used as an enrichment biomarker in upcoming clinical trials in prodromal synucleinopathies.^22^ Moreover, in a recent proof‐of‐concept study, we showed that ^123^I‐FP‐CIT‐SPECT may also be used as a progression biomarker for disease modifying therapies.^23^ It is, at the moment, the best second‐line stratification tool, but it is likely not suitable as a first‐line stratification tool for selecting prodromal PD patients to be enrolled in clinical trials because it is expensive, not available at all sites, and it exposes patients to radiation.

In the present study, we found that cognitive function, in particular attention and working memory, is a valuable predictor of short‐term phenoconversion in iRBD patients, with the TMT‐B achieving a sensitivity of 100% when a cut‐off of 85 seconds was used. When planning a disease‐modifying clinical trial, a well‐known, sensitive, and cost‐effective stratification tool is needed. Such tool should be usable in a large number of eligible iRBD patients with the aim of reducing the number of subjects at risk that will undergo the second‐line stratification tool (ie, ^123^I‐FP‐CIT‐SPECT), excluding the iRBD patients that will likely be non‐converters. The TMT‐B would reduce the number of eligible subjects by ~23%, but ensuring that no short‐term phenoconverter is lost. Subsequently, if a ^123^I‐FP‐CIT ‐SPECT cut‐off of −1 standard deviation would be used, the sample would be reduced by a further 11% (to 66% of the original sample), ensuring a high likelihood of retaining virtually all phenoconverters (see Fig. 2 for details). Moreover, this approach ensures good sensitivity (0.88). For comparison, if hyposmia would be used as a first‐line stratification tool and ^123^I‐FP‐CIT ‐SPECT cut‐off of −1.5 standard deviation, at putaminal level, were chosen as second‐line stratification tool, the number of eligible subjects would drastically decrease to 25%, but with low sensitivity (0.41) and good specificity (0.83).

Cognitive impairment has been consistently found as a significant risk factor of phenoconversion in iRBD patients.^1^, ^2^, ^20^, ^24^ In particular, the TMT‐B has been already proposed as an early diagnostic test for inclusion in clinical trials.^25^ Moreover, cognitive impairment is often seen in PD patients in the early stages, mainly of the dysexecutive type, and 20% to 25% of PD patients already have MCI at diagnosis,^16^, ^26^ therefore, it is not surprising that TMT‐B is a good predictor of phenoconversion in prodromal synucleinopathies for both PD and DLB phenoconversion diagnosis. A recent study showed that cerebrospinal fluid investigation of α‐synuclein, by means of real‐time quaking‐induced conversion (RT‐QuIC) technology, may be an excellent tool for identifying iRBD patients that will not likely develop a full‐blown neurodegenerative disease, at least not within 7 to 10 years from baseline.^27^ However, the lumbar puncture is invasive, hardly feasible as first‐line stratification tool, and larger, independent studies are needed to validate such a promising technique.

To investigate whether baseline risk factors may be able to predict the phenoconversion diagnosis (ie, PD vs. DLB), we conducted a ROC analysis, showing that DLB‐converters had more severe baseline clinical, neuropsychological, and instrumental features, compared with PD‐converters. We found that DLB‐converters had significantly reduced executive function, prominent EEG slowing in posterior regions, and a more severe nigrocaudate dopaminergic deafferentation compared with PD‐converters, in agreement with literature data. Indeed, executive functions are often impaired in both PD and DLB patients,^28^ but in iRBD patients eventually developing DLB, the cognitive impairment is expected to be earlier and more severe than in iRBD patients eventually developing PD. DLB patients have typical EEG abnormalities, and prominent posterior slow‐wave activity on EEG have been included as supportive biomarkers for the DLB diagnosis.^11^ Finally, nigrocaudate dopaminergic impairment is usually more severe in DLB than in PD patients.^29^ These results are also in agreement with those of a recent, large multicentric study showing cognitive impairment and nigrocaudate dopaminergic deafferentation as the most informative biomarkers able to predict the phenoconversion diagnosis (ie, DLB vs. PD).^2^ Finally, it has to be highlighted that EEG posterior slowing and nigrocaudate deafferentation are significantly correlated with each other in phenoconverted patients only, therefore, suggesting that the value in prediction phenoconversion diagnosis of these two variables may be relevant only in subjects at high risk of phenoconversion.

As a final comment, we highlight that we conducted a preliminary analysis to investigate neuropsychological, EEG, and ^123^I‐FP‐CIT‐SPECT characteristics of iRBD patients, compared with healthy subjects, to better characterize our patients. This is a crucial step for the interpretation of the longitudinal results. For example, here, we confirm previous findings showing that verbal memory and visuoconstruction domains are frequently impaired in iRBD patients.^30^ However, from a prediction point of view, the TMT‐B is more informative, perhaps because it is not extensively impaired in all patients at baseline, therefore, expressing more variance in a longitudinal design. Moreover, even if we confirm that iRBD patients have significant EEG slowing comparing with healthy subjects,^31^ in the longitudinal analysis this feature is mainly relevant for the prediction of DLB‐converters versus PD‐converters. Finally, we confirm previous data showing that iRBD patients have significant nigrostriatal dopaminergic impairment compared with healthy subjects, but the nigroputaminal data are the most relevant for phenoconversion prediction.^2^

The main strength of the study is that for the first time a multivariate analysis including clinical, brain functional imaging, neuropsychological, and neurophysiological markers were explored to identify first‐ and second‐line screening tools for iRBD patients' stratification, in preparation for the upcoming disease‐modifying trials in prodromal synucleinopathy. The main limitation of the study is that it was conducted in a single center, with limited number of subjects. However, this ensured robust and harmonized analysis of the data, which is mandatory for obtaining reliable data, especially for brain imaging techniques. Nevertheless, the present data, in particular sensitivity and specificity should be validated in an independent sample. Another limitation of the study is that the controls did not undergo polysomnography.

In conclusion, the present results suggest that the TMT‐B may be used as an easy, cost‐effective, and fast first‐line stratification tool, followed by ^123^I‐FP‐CIT‐SPECT as a second‐line stratification tool, to be used to select eligible iRBD patients to be enrolled in upcoming disease‐modifying clinical trials.

## Author Roles

(1) conception and design of the study.

(2) acquisition and analysis of data.

(3) drafting a significant portion of the manuscript or figures.

D.A.: 1, 2, 3

P.M.: 2, 3

F.F.: 2

N.G.: 2

A.B.: 2

M.P.: 2, 3

A.D.: 2

F.M.: 2

B.O.: 2

S.R.: 2

M.B.: 2

S.M.: 2, 3

F.N.: 1, 2, 3

## Supporting information


**Appendix S1**: Supporting Information

## Data Availability

The data that support the findings of this study are available from the corresponding author upon reasonable request.
