# Supplementary material for: Stratification Tools for Disease‐Modifying Trials in Prodromal Synucleinopathy
Source: Mov Disord. 2021 Sep 17;37(1):52–61. doi: 10.1002/mds.28785 (PMC9292414; doi:10.1002/mds.28785)
Supplement: Supplementary file 1 — Appendix S1: Supporting Information [file MDS-37-52-s001.docx]

Supplementary Table 1. Hazard ratio (HR) (and relative 95% confidence intervals, CI) and area under the curve (AUC) values of the explored variables, ordered by the HR values.

| **Category** | **Variable** | **HR** | **95% CI** | **AUC** |
| --- | --- | --- | --- | --- |
| DAT SPECT | Putamen SBR | 7.28 | 1.80-29.42 | 0.77 |
| NPS | AT/WM | 5.91 | 1.78-19.67 | 0.84 |
| DAT SPECT | Caudate SBR | 4.36 | 1.34-14.21 | 0.70 |
| EEG | Occipital MF | 2.80 | 1.01-7.76 | 0.68 |
| EEG | Centro-Parietal ratio | 2.76 | 0.99-7.64 | 0.64 |
| EEG | Occipital ratio | 2.72 | 0.96-7.69 | 0.63 |
| EEG | Temporal ratio | 2.71 | 0.96-7.67 | 0.68 |
| EEG | Frontal ratio | 2.48 | 0.86-7.15 | 0.59 |
| NPS | VM | 2.38 | 0.87-6.47 | 0.57 |
| Clinical | MDS-UPDRS-III | 2.28 | 0.84-6.17 | 0.71 |
| Clinical | MCI | 1.98 | 0.71-5.38 | 0.61 |
| EEG | Centro-Parietal MF | 1.91 | 0.65-5-62 | 0.64 |
| Clinical | Orthostatic Hypotension | 1.47 | 0.41-5.25 | 0.57 |
| NPS | VS | 1.46 | 0.51-4.19 | 0.69 |
| EEG | Frontal MF | 1.31 | 0.47-3.64 | 0.56 |
| Clinical | Constipation | 1.27 | 0.45-3.61 | 0.62 |
| EEG | Temporal MF | 1.23 | 0.40-3.74 | 0.62 |
| NPS | EX | 1.07 | 0.38-3.03 | 0.58 |
| Clinical | Hyposmia | 0.82 | 0.30-2.18 | 0.52 |

Legend: AT/WM, attention and working memory; DAT, dopamine transporter; EX, executive functions; MCI, mild cognitive impairment; MDS-UPDRS-III, MDS revision of the unified Parkinson’s disease rating scale, motor section; MF, Mean Frequency; NPS, neuropsychology; SBR, Specific to non-displaceable binding ratio; VM; verbal memory; VS, Visuo-spatial.

Supplementary Table 2. AUC values of explored variables for the differentiation of PD converters vs DLB converters.

| **Category** | **Variable** | **AUC** | **Worse in** |
| --- | --- | --- | --- |
| EEG | Centro-Parietal MF | 0.81 | DLB |
| NPS | EX | 0.81 | DLB |
| EEG | Temporal MF | 0.79 | DLB |
| EEG | Occipital MF | 0.75 | DLB |
| DAT SPECT | Caudate SBR | 0.71 | PD |
| EEG | Temporal ratio | 0.71 | DLB |
| EEG | Occipital ratio | 0.69 | DLB |
| EEG | Frontal ratio | 0.65 | DLB |
| DAT SPECT | Putamen SBR | 0.64 | PD |
| EEG | Centro-Parietal ratio | 0.64 | DLB |
| Clinical | Orthostatic Hypotension | 0.63 | PD |
| NPS | VM | 0.56 | DLB |
| Clinical | Hyposmia | 0.53 | DLB |
| Clinical | MCI | 0.53 | DLB |
| Clinical | Constipation | 0.52 | DLB |
| NPS | AT/WM | 0.51 | DLB |
| NPS | VS | 0.51 | PD |
| Clinical | MDS-UPDRS-III | 0.51 | DLB |
| EEG | Frontal MF | 0.49 | DLB |

Legend: AT/WM, attention and working memory; DAT, dopamine transporter; EX, executive functions; MCI, mild cognitive impairment; MDS-UPDRS-III, MDS revision of the unified Parkinson’s disease rating scale, motor section; MF, Mean Frequency; NPS, neuropsychology; SBR, Specific to non-displaceable binding ratio; VM; verbal memory; VS, Visuo-spatial.
